# Polymer Coated Urea in Turfgrass Maintains Vigor and Mitigates Nitrogen's Environmental Impacts

**DOI:** 10.1371/journal.pone.0146761

**Published:** 2016-01-14

**Authors:** Joshua J. LeMonte, Von D. Jolley, Jeffrey S. Summerhays, Richard E. Terry, Bryan G. Hopkins

**Affiliations:** 1 Department of Plant and Soil Sciences, University of Delaware, Newark, Delaware, United States of America; 2 Plant and Wildlife Sciences Department, Brigham Young University, Provo, Utah, United States of America; Institute for Sustainable Plant Protection, C.N.R., ITALY

## Abstract

Polymer coated urea (PCU) is a N fertilizer which, when added to moist soil, uses temperature-controlled diffusion to regulate N release in matching plant demand and mitigate environmental losses. Uncoated urea and PCU were compared for their effects on gaseous (N_2_O and NH_3_) and aqueous (NO_3_^-^) N environmental losses in cool season turfgrass over the entire PCU N-release period. Field studies were conducted on established turfgrass sites with mixtures of Kentucky bluegrass (*Poa pratensis* L.) and perennial ryegrass (*Lolium perenne* L.) in sand and loam soils. Each study compared 0 kg N ha^-1^ (control) to 200 kg N ha^-1^ applied as either urea or PCU (Duration 45CR®). Application of urea resulted in 127–476% more evolution of measured N_2_O into the atmosphere, whereas PCU was similar to background emission levels from the control. Compared to urea, PCU reduced NH_3_ emissions by 41–49% and N_2_O emissions by 45–73%, while improving growth and verdure compared to the control. Differences in leachate NO_3_^-^ among urea, PCU and control were inconclusive. This improvement in N management to ameliorate atmospheric losses of N using PCU will contribute to conserving natural resources and mitigating environmental impacts of N fertilization in turfgrass.

## Introduction

Nitrogen is an essential plant nutrient in the biosphere. Conversion or fixation of the ubiquitous pool of atmospheric N_2_ gas to biologically active amine (RNH_2_) forms occurs through both abiotic (lightning, combustion, and Haber-Bosch industrial processes) and biotic (microbial and plant) processes. These processes are essential for life and lead to biosynthesis of nucleotides used for deoxyribonucleic acid (DNA) and ribonucleic acid (RNA) synthesis and amino acids for protein/enzyme production. Although background levels of natural fixation enable plants to grow in the wild, N fertilization is required to provide adequate food, fuel, and fiber to sustain the ever-growing human population.

Despite its benefits and essentiality for life, N is also a common pollutant in the biosphere. Annual worldwide N fertilizer application is projected to total 139 million metric tons in 2011/2012 [1]. Ideally, applied N is held in the soil until absorbed by plants but may just as likely be evolved as ammonia (NH_3_) or nitrous oxide (N_2_O) gases or be leached or run off as nitrate (NO_3_^-^).

It is estimated that 10% of manufactured N fertilizer worldwide is volatilized as NH_3_ gas [2]. In a growth chamber study using warm-season bentgrass (*Agrostis palustis* Huds.), volatilization of surface-applied N fertilizers was in excess of 60% over the first 10 days following surface application [3]. Researchers at Kansas State University found nearly 100% loss of N applied to maize (*Zea mays* L.) from a broadcast liquid urea under worst-case conditions of high temperature (>30°C), humidity (>95% RH), and wind (>30 km hr^-1^) (Bryan Hopkins, unpublished data, 2011). Volatilized NH_3_ gas is a serious environmental concern because it contributes to photochemical smog [4] and is more likely to be deposited on land or water bodies than other forms of anthropogenic N. Ammonia deposition in sensitive ecosystems can lead to soil acidification [5] and surface water eutrophication [6]. Nitrogen deposition can also lead to plant community loss and reduction of biodiversity [5]. Increased N availability from NH_3_ deposition in typically N-limited ecosystems is leading to unwanted consequences across the globe including increased aluminum mobility resulting in forest decline [7].

Elevated emissions of N_2_O are also concerning as an estimated 1% of N applied in inorganic forms is lost to the atmosphere as N_2_O [8]. The actual amount lost varies and is directly related to the type, quantity, and method of application of the applied fertilizer [8]. It is estimated that anthropogenic emissions of N_2_O have increased by approximately 50% over pre-industrial levels [9]; fertilization accounts for 78% of that total, with automobile and industrial pollution making up most of the remainder [10]. The environmental concerns with N_2_O are that it is a long-lived, potent greenhouse gas with a global warming potential 310 times greater than carbon dioxide (CO_2_) [10] and it catalytically destroys ozone (O_3_) in the troposphere [11]. The net effects are increased warming potential and more UV radiation exposure to living organisms. Emissions of N_2_O to the atmosphere via denitrification and nitrification are controlled by many interacting factors including soil aeration, temperature, texture, ammonium (NH_4_^+^), nitrite (NO_2_^-^), and nitrate (NO_3_^-^)-N concentrations, and microbial communities [12], [13].

Although N losses to the atmosphere are disconcerting and currently dominate public forums, losses to the hydrosphere are also a serious concern [14]. Soil NO_3_^-^ easily leaches below the rooting zone due to similar negative ionic charges of soil particles and the NO_3_^-^-N, resulting in increased N in ground waters. Nitrate (and other forms of N) can also be surface transported through runoff and erosion, resulting in increased N in surface waters. The amount of N lost is a function of fertilizer source, timing, soil infiltration and percolation rate, micropore flow, root density, soil moisture, and precipitation/irrigation rate and intensity [15], [16]. In addition to the decreased plant available N that results, excess NO_3_^-^ in watersheds can lead to toxicological problems such as eutrophication (large algal blooms which can lead to anoxic conditions) and drinking water contamination [17]. Drinking water contaminated with NO_3_^-^ is thought to cause methemoglobinemia (blue baby syndrome) in young animals and human babies [15]. Excess NO_3_^-^ in watersheds may also be toxic to freshwater biota and disrupt nutrient cycling [7].

In addition to the environmental impacts of excess N, manufacturing N fertilizer uses natural gas and other non-renewable resources. Thus, minimizing N losses and maximizing plant utilization are critical to conserving non-renewable resources and environmental quality. Effectiveness of N uptake and utilization by plants is defined as nitrogen use efficiency (NUE) [18].

Optimizing N fertilizer rate, source, timing, and placement are all essential to maximizing NUE [18]. The correct N rate is vital for balancing optimal plant growth and minimal residual soil N. Timing of N application is important so as to match N uptake periods with N delivery and minimizing available soil N when plants are less active. Placement is also important so that N is found in the active root zone—for turfgrass it is essential to retain the N in the upper soil profile for this shallow rooted species.

Additionally, there are many N sources and additives to choose from. One tactic to increase NUE is to use controlled-release N (CRN) or slow-release N (SRN). These sources release N into the soil over an extended, specified period of time to ideally match plant needs and possibly to reduce or eliminate labor-intensive, costly in-season N applications [18]. By controlling the release of N from fertilizer, N inefficiencies and losses to the environment should be mitigated via increased N retention by the soil and uptake by the plant [12], [18]. The concept of CRN and SRN fertilizers is not new, but success has varied across plant species and environmental conditions and expense has prevented wide utilization [18]. More recently, cost of these products has become more competitive with traditional N sources.

Polymer-coated urea (PCU) is one promising type of CRN fertilizer that provides improved N-release timing. Soil temperature controls N release rate from certain PCU, which allows protection of N during cool periods when plants are not growing and soils are often susceptible to N losses, but then release of N as temperatures improve and plant growth and N uptake increase [18]. Diffusion of N through the polymer coating is driven by an N concentration gradient—temperature being the primary regulator under irrigated conditions. Some PCU sources steadily supply plants with N for longer periods of time following application than immediately soluble forms of N, thus enhancing NUE [18], [19], [20], [21], and leading to increased crop yield and quality [3], [22], [23], [24], [25], [26]. Hyatt et al. (2010) showed that the slower release of PCU can improve economics by eliminating additional in-season N applications in potato [27].

Research has also demonstrated PCU’s ability to mitigate negative environmental impacts associated with N fertilizer [21], [28], [29]. Polymer-coated urea was shown to decrease NO_3_^-^ leaching [21], [23], [28], [30], [31], [32], NH_3_ volatilization [3], [33], [34] and N_2_O emissions [27], [29], [35], [36], [36], [37]. However, there have also been studies that have observed no decrease N loss compared to urea [38], [39].

Most work investigating anthropogenic inputs to the atmosphere from fertilization has been performed in intensive row crop agricultural systems [such as maize, wheat (*Triticum* spp.), and potato (*Solanum tuberosum* L.)]. Very little has been done in grass systems [3], [37] despite N fertilizers having a major role for urban turfgrass and agricultural sod, seed, and pasture grass systems. Turfgrass occupies 1.9% of the total surface area of the United States and is the leading irrigated crop in the country [40]. Agrarian turfgrass landowners apply between 75 and 500 kg N ha^-1^ as fertilizer each year [40]. These rates, though comparable to the most intensively cultivated agriculture fields in the world, are often exceeded by urban homeowner applications. Nowhere is the attitude of “if a little is good then more is better” more prevalent than with those applying N to turf, especially when visual greening is so apparent. Additionally, homeowners are often uneducated in regard to appropriate rates of fertilizer and methods of correct application. The wide geographical distribution and excessive N application by homeowners and turfgrass managers can lead to environmental and economic concerns.

The primary objective of this study was to compare NH_3_ and N_2_O gas evolution, NO_3_^-^ leaching and plant uptake and verdure on a mixture of Kentucky bluegrass (KBG; *Poa pratensis* L.) and perennial ryegrass (PRG; *Lolium perenne* L.) turfgrass treated with polymer-coated urea, urea, and untreated control.

## Materials and Methods

Two field studies were conducted on property owned by Brigham Young University (BYU) after obtaining specific permission through the Department of Plant and Wildlife Sciences. These studies, located in Utah, USA, were conducted on established turfgrass, with a mixture of Kentucky bluegrass (KBG; *Poa pratensis* L.) and perennial ryegrass (PRG; *Lolium perenne* L.). Best management practices for growing cool season turfgrass were generally used at both sites. Site 1 in Provo (40°16’1.40”N 111°39’28.59”W) is a sports turfgrass sod farm at BYU with sandy soil (Table 1). Site 2 near Spanish Fork (40°4’1.77”N 111°37’44.99”W) is a turfgrass area at the former BYU experimental research center with a Timpanogos loam soil and located adjacent to a weather station (Table 1). At each site, 1 m x 3 m plots were established immediately next to each other in a randomized complete block design with three treatments and six replications. Treatments included application of 0 (control) or 200 kg N ha^-1^ applied as either urea (46-0-0) or polymer-coated urea (PCU or Duration 45 CR®, Agrium Advanced Technologies, Loveland, CO). Treatments were uniformly surface applied on DOY 271 and 285 of 2008 for sites 1 and 2, respectively.

**Table 1:** Selected characteristics of soils on which two field studies were conducted on established, mixed stands of Kentucky bluegrass and perennial ryegrass turf.

Site^†^	Location (in UT)	Texture	NO3^-^-N	OM	pH	Sand	Silt	Clay
			- ppm -	- % -		- % -	- % -	- % -
1	Provo	Sand	2.67	0.77	6.93	87.64	4.20	8.16
2	Spanish Fork	Loam	3.68	2.80	7.17	52.00	25.56	22.44

^†^Site 1 is a sport’s turfgrass sod farm at Brigham Young University, Provo, UT on a disturbed sand soil and Site 2 is at the former BYU Research Station, Spanish Fork, UT on a Timpanogos loam soil.

Plots were irrigated with approximately 2 cm water within 12 and 1 h after fertilizer application for sites 1 and 2, respectively. Irrigation was managed by monitoring soil volumetric water content using Watermark Soil Moisture Sensors (Spectrum Technologies, Plainfield, Illinois, USA) and logged using an AM400 soil moisture data logger (MK Hansen, Wenatchee, Washington, USA). Soil temperature was monitored with a thermistor and logged using the same data logger. Weather was typical for this region with daily temperatures averaging 16.5°C max and 2.8°C min with a general trend of decreasing temperatures during the trials. Total rainfall for the experiment was 99 mm at Site 1 and 113 mm at Site 2.

Simplified modified passive flux collection devices were installed near the center of each plot to collect volatilized NH_3_. Passive flux sampling tubes were vertically oriented (to minimize cross plot gas contamination) with the bottom of each tube 10 cm above the plant-soil interface. Each sampling device consisted of a glass tube (0.7 cm inside diameter x 10 cm length), with the interior coated with 3% w/v oxalic acid in acetone to readily react with and collect NH_3_ from the air flowing through the tube. Flux tubes were replaced daily for the first two days, then every three to four days thereafter for an additional 21d (until volatilization levels returned to ambient conditions) and then weekly thereafter. When collected for analysis, tubes were capped immediately with rubber septa stoppers to eliminate potential contamination. The NH_3_ was extracted from the flux tubes by adding 1 ml of deionized water, recapping with septa stoppers and shaking mechanically for 10 min using a modified vortex mixer (Labnet International, Inc., Woodbridge, NJ, USA). Extracts were then diluted with 2 ml of deionized water and analyzed for NH_4_^+^ using the automated cadmium reduction method [41] with a Lachat colorimetric analyzer (Lachat Instruments, Loveland, CO, USA). Analytical results were expressed as total NH_3_-N (mg). These values were converted to flux by implementing principles of the ideal gas law (PV = nRT) at standard temperature and pressure. Flux was calculated by

$$ƒ=\frac{C}{A\times t}$$

Where *C* is the concentration of NH_4_^+^ as measured by the Lachat, *A* is the area of the collection tube, and *t* is the time the flux tube was exposed to the atmosphere.

Vented poly-vinyl chloride (PVC) static (18 cm diameter x 28 cm height) collection chambers were installed near the center of each plot to collect N_2_O gas. These PVC collars were fitted with rubber gaskets on the top lip and buried to a depth of 6–8 cm into the soil. During periodic sampling times, the chamber lid was attached to the top of each chamber and sealed with a rubber gasket. Samples were taken three days a week for the first 28 d and 22 d (Sites 1 and 2, respectively) after fertilizer application, and once or twice a week thereafter. Samples were taken through a septum on top of the chamber with a 10-ml glass syringe fitted with a rubber stopper at intervals of 15, 30, and 45 min after installing the lid to the chamber. Samples were immediately transported to the laboratory and analyzed within 4–6 h with a gas chromatograph coupled with an electron capture detector (GC, Agilent 6890N, Agilent Technologies, Santa Clara, California, USA) [42]. Flux was determined as described by Mosier et al. (1991) [43] and by implementing principles of the ideal gas law (PV = nRT) at standard temperature and pressure. Flux was calculated by

$$ƒ=\frac{V\times \Delta C}{A\times \Delta t}$$

Where *V* is the headspace volume, *ΔC* is the change in concentration [C_o_ (initial concentration)—C_t_ (concentration at time t)] of N_2_O as measured by the GC, *A* is the area of the static chamber, and *Δt* is the time elapsed between C_o_ and C_t_.

Suction lysimeters (24 in. 1900 Series, Soil Moisture Corp., Goleta, CA, USA) were installed in three blocks at each site at a 30° angle to a depth of 8–10”. Leachate was collected and NO_3_^-^ N concentrations determined using a Lachat automated analyzer for NO_3_^-^ N by the salicylate nitroprusside method [41]. Soil NO_3_^-^ N samples were taken 21 and 45 d after application from 0–30 and 30–60 cm depths and concentrations were analyzed as described above.

Root and shoot samples were also taken 21 and 45 d after fertilization and dried at 65°C, ground and analyzed for total N (Leco® TruSpec® CN Elemental Determinator, St. Joseph, MI, USA). Verdure assessments were done via visual ratings on a 1–5 scale, with 1 being brown and 5 being dark green.

All data (S1 Dataset) was analyzed for statistical significance by ANOVA and Tukey HSD analyses using JMP 10.8 statistical software to compare all possible pairs (SAS Institute, Cary, NC, USA). Prior to analysis, data was checked for normality and those data sets that were not normally distributed were log transformed and analyzed for significance. Significance levels were *P* < 0.05.

## Results and Discussion

### Ammonia

**Fig 1:**
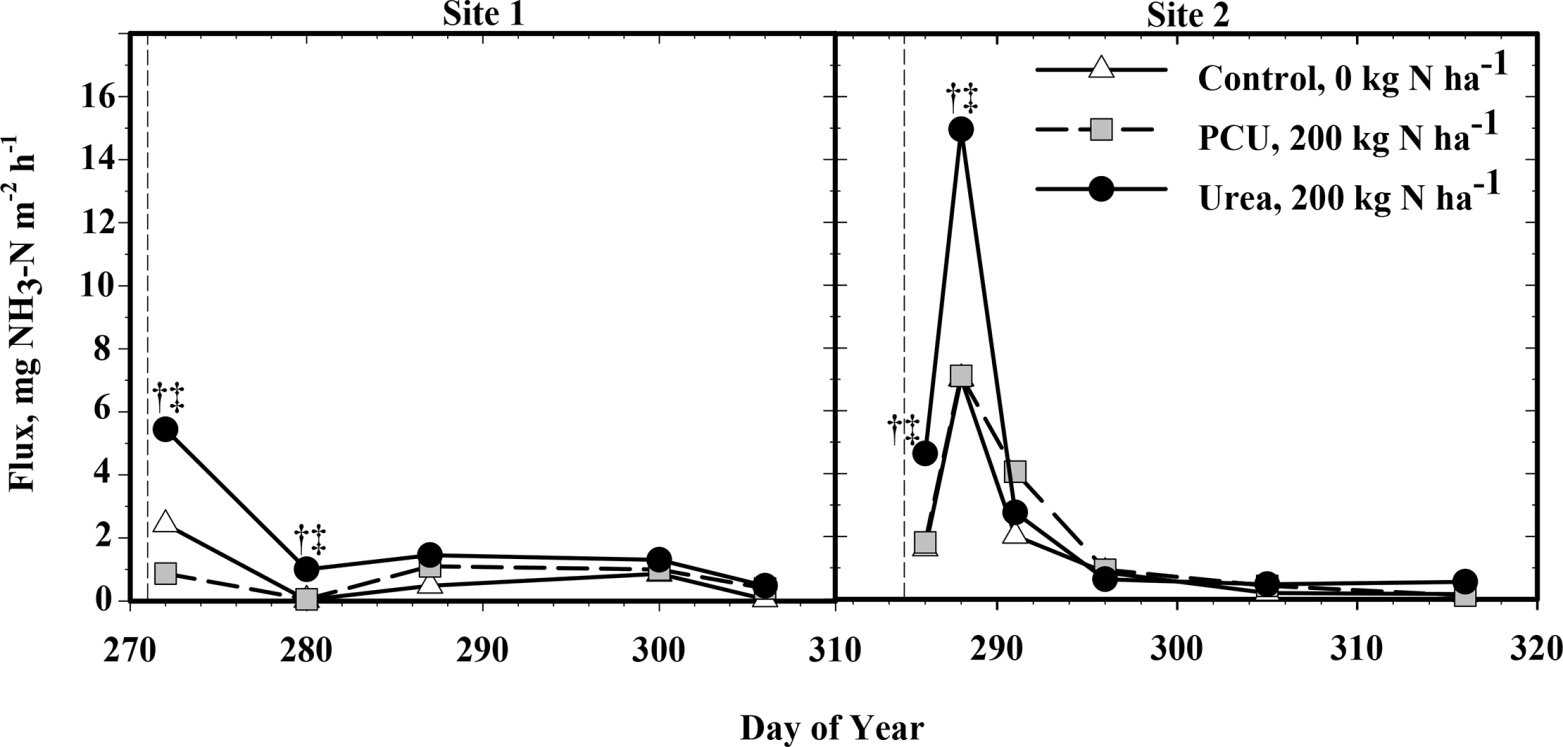
Passive flux of NH_3_-N measured on multiple individual days for Sites 1 and 2 from application of 0 (Control) and 200 kg N ha^-1^ polymer coated urea (PCU) or urea. Significance indicated by † (urea greater than control) and ‡ (urea greater than PCU) where *P* = 0.05. Vertical dashed lines indicate fertilizer application.

Ammonia volatilization from the urea application at Site 1 was significantly higher than the control and PCU on two sampling days (DOY 272 and 280; Fig 1; *P =* 0.05). Ammonia volatilization from the PCU application was never higher than the control or urea during any sampling event (Fig 1). Ammonia volatilization reached a peak from both urea and PCU one day after fertilization (DOY 272). At Site 2, NH_3_ volatilization from the urea application was significantly higher than the control and PCU on two of the six sampling dates, similar to Site 1 (Fig 1; DOY 286 and 288; *P =* 0.05). In contrast, PCU never produced significantly greater NH_3_ volatilization than the control or urea during any sampling event. Ammonia volatilization with urea and PCU reached a peak three days after fertilization (DOY 288). This flush of NH_3_ volatilization shortly after fertilization is typical for surface applied N fertilizers. The difference in magnitude of measured volatilization can be attributed to the difference in soil types [34].

**Fig 2:**
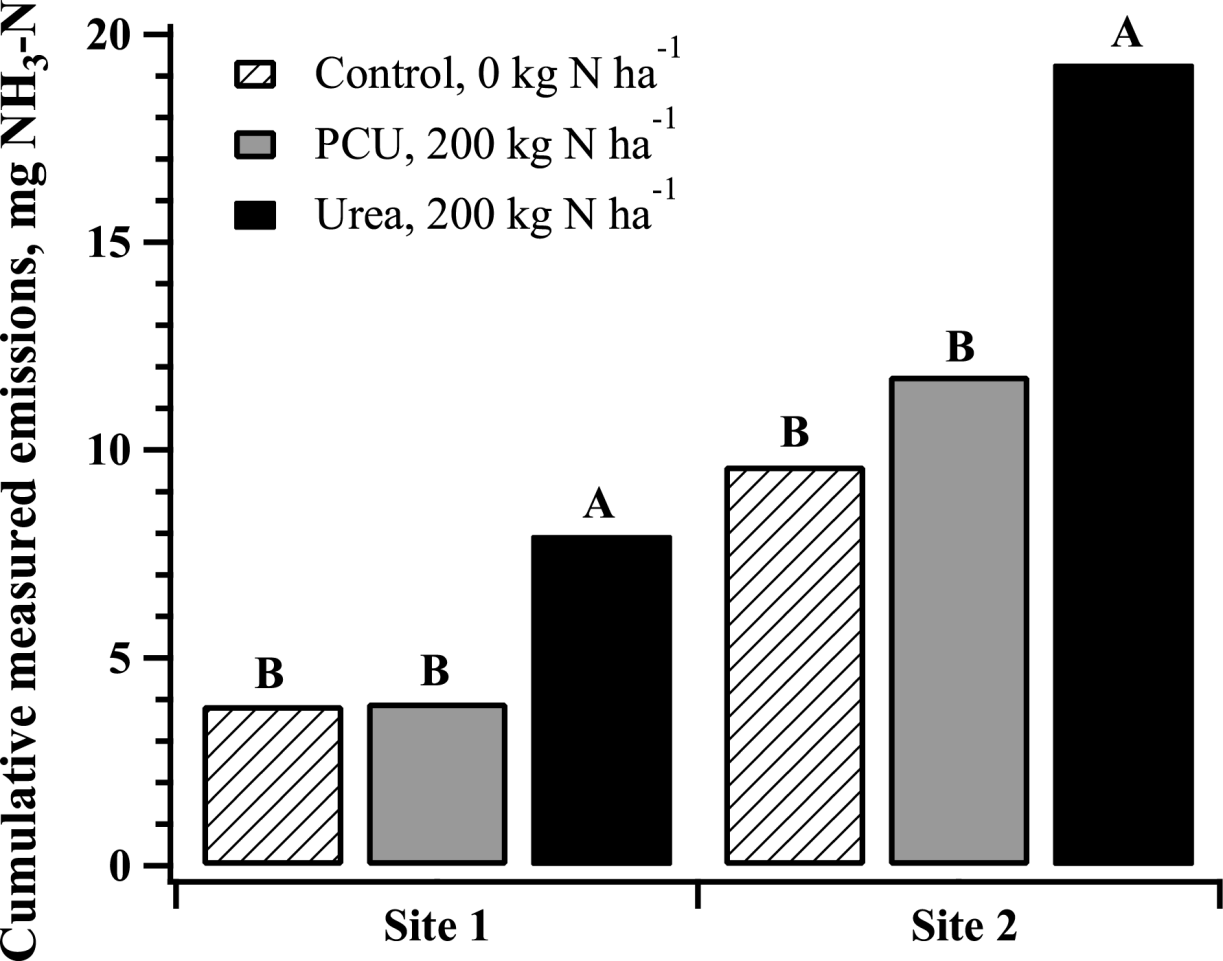
Cumulative NH_3_ emissions over a period of 45 days after application for two field sites from 0 (Control) and 200 kg ha^-1^ N polymer coated urea or urea. Sources for a given site with the same letter are not significantly different at *P* = 0.05, Tukey HSD.

Cumulative NH_3_ volatilization trends were similar at both sites (Fig 2). Statistically similar relative amounts of NH_3_ were volatilized from the control and PCU, while significantly more NH_3_ volatilization was measured from the urea application than from the control and PCU (*P* = 0.04 and 0.07, respectively; Fig 1). Thus, using PCU as N fertilizer decreased relative NH_3_ volatilization by 51% and 39% for site 1 and 3, respectively, compared to urea.

The relative reductions of NH_3_ gas loss found in this study attributable to PCU are similar to those reported in another study with turfgrass [3] and in maize systems [33]. Knight et al. (2007) conducted growth chamber experiments with a warm season turfgrass species, ‘G-2’ creeping bentgrass (*Agrostis stolonifera* L.), grown in a loamy sand and found that PCU had the lowest levels of NH_3_ volatilization among the six forms of N fertilizers tested, with no noticeable spike in volatilization in the 10 d length of the study [3]. However, the PCU used in that study would have been expected to release its N over a longer period of time than 10 d. Our study was conducted over the entire 37 to 42 days of anticipated N release from the PCU and no spike in the NH_3_ volatilization was observed in the late season. The pattern of NH_3_ volatilization from PCU follows that of the control, with no significant differences measured. Overall, our data support the findings of Knight et al. (2007), inasmuch as PCU significantly decreases NH_3_ emissions when compared to urea.

### Nitrous Oxide

**Fig 3:**
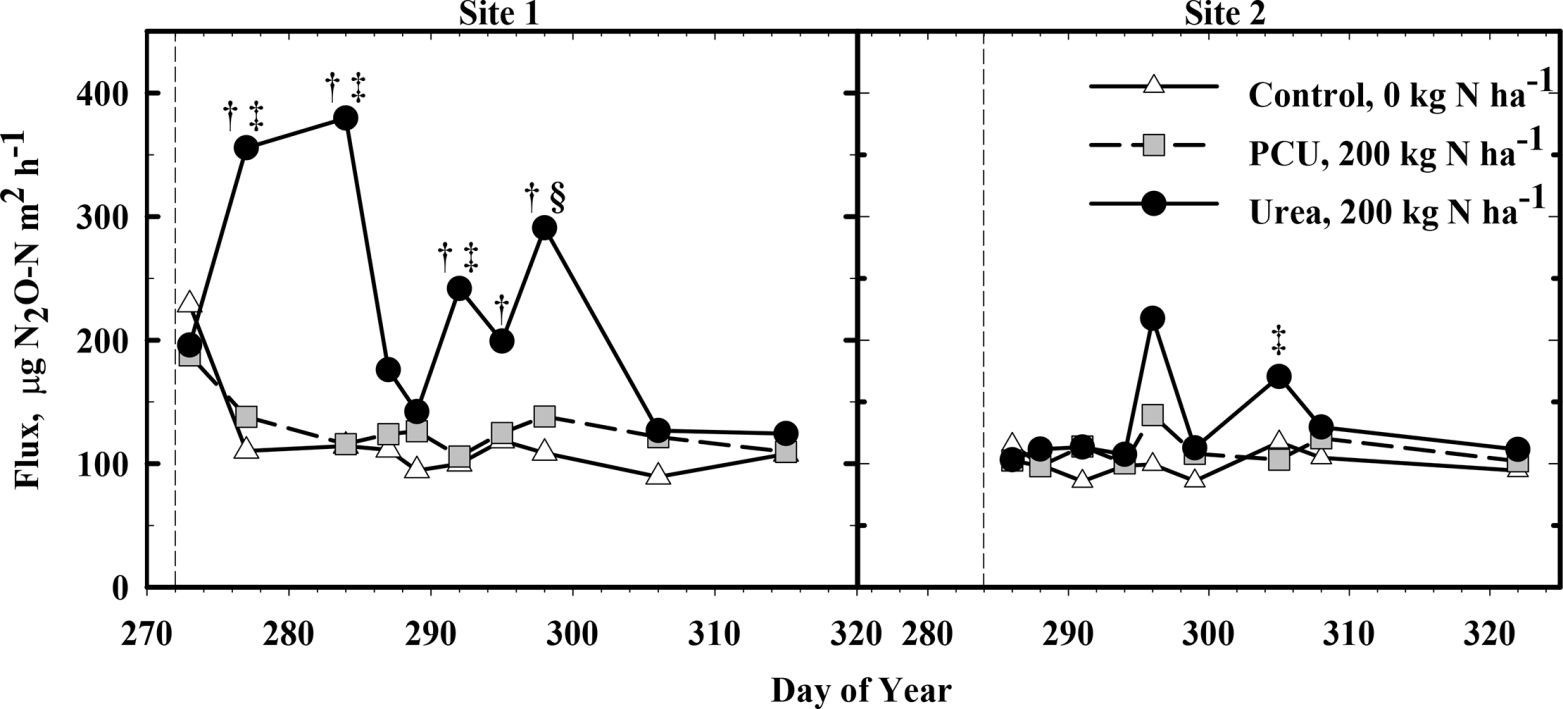
Nitrous oxide (N_2_O) flux measured on multiple individual days for Sites 1 and 2 from application of 0 (Control) and 200 kg N ha^-1^ polymer coated urea (PCU) or urea. Significance for comparing fertilizer source on a given day is indicated by † (urea greater than control), ‡ (urea greater than PCU), and § (PCU greater than control). Vertical dashed lines indicate date of fertilizer application at a given site.

The N_2_O flux at site 1 from the urea application were significantly higher than the control on five of the 10 sampling dates (Fig 3; *P =* 0.05). In contrast, PCU produced significantly greater N_2_O flux than the control on only two of 10 sampling periods. When comparing sources, N_2_O emissions from urea were significantly higher than PCU on two sampling days, and PCU never produced significantly higher N_2_O flux than urea. Flux of N_2_O from plots treated with urea peaked approximately five days after fertilization (DOY 277) and from plots receiving PCU approximately 26 days (DOY 298) after fertilization. There were fewer observations of differences among treatments at site 2; i.e. one d on which N_2_O emissions from urea were greater than control at the *P* = 0.07 level (DOY 309) and one on which emissions were greater than PCU (DOY 307, Fig 3). Nitrous oxide emission from PCU never exceeded N_2_O flux from urea.

**Fig 4:**
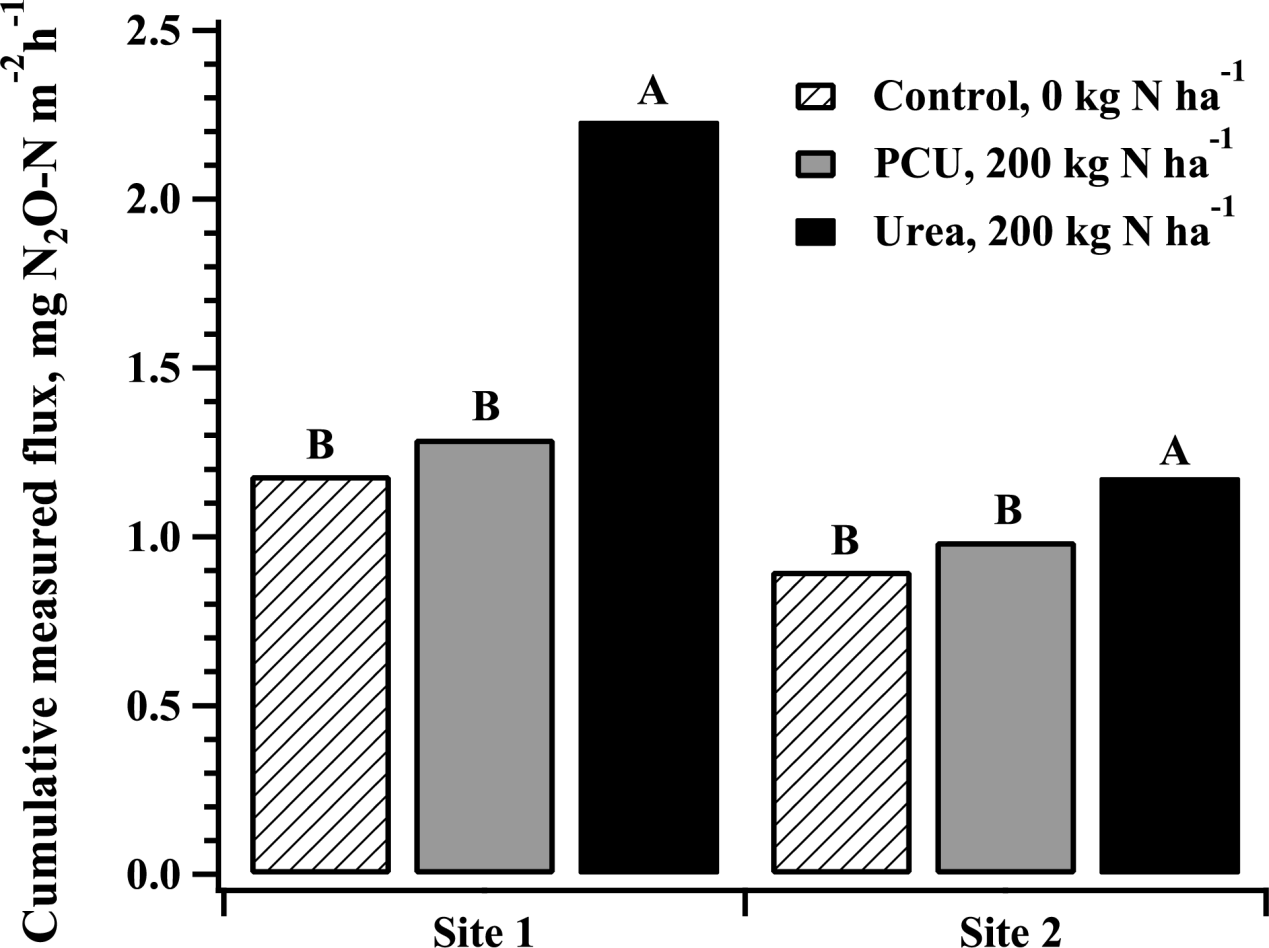
Cumulative N_2_O emissions measured over a period of 45 days after application for two field sites from 0 (Control) and 200 kg ha^-1^ N polymer coated urea or urea. Sources for a given site with the same letter are not significantly different at *P* = 0.05, Tukey HSD.

Cumulative measured N_2_O emissions were calculated by summing the actual values (mg N_2_O m^-2^) obtained over the 10 days sampled and therefore do not represent a total cumulative loss but a relative representation of N_2_O losses over the course of the trial. For site 1, urea produced 3.9 and 2.1 times the N_2_O produced by the control and PCU, respectively (*P* < 0.0001 for both), while control and PCU emissions were statistically similar (Fig 4; P = 0.761). The actual emissions for control, PCU and urea were 5.6, 10.5, and 22.1 mg m^-2^, respectively.

Despite fewer d of observed differences among treatments at site 2, relative total N_2_O emission were in the same order as at site 1 (Fig 4). The total measured flux from urea treatments at site 2 was 1.8 times that of PCU and 2.3 times that of the control (*P* = 0.001 and 0.026). Actual sum of emissions for control, PCU and urea were 21.6, 23.6 and 28.2 mg m^-2^ respectively. As at site 1, relative total emission was statistically similar for PCU and control (*P* = 0.31, Fig 4).

Difference in water management and consequential soil moisture differences at each site likely contributed to the variation of intensities of N_2_O flux seen at these two sites. Site 1 was irrigated daily (as it had a sand texture, albeit with a perched water table at about 10 cm below the soil surface) and likely maintained a somewhat high soil moisture level (3.5% volumetric water content fluctuations), while the Site 2 was irrigated approximately weekly, soil moisture likely fluctuated widely (10.9% volumetric water content fluctuations) and overall moisture averaged 5% lower than Site 1. Although fine-textured soils, which comprise the soil at Site 2, typically favor N_2_O emissions via denitrification [44], soil moisture levels were mostly maintained below the anaerobic threshold with the soils being saturated at irrigation and then allowed to dry down for ~7 d, thereby promoting nitrification. Site 1 also avoided saturation for longer than a few minutes daily because of the nature of the sand-based system employed. Thus, the source of N_2_O emission under these aerobic, slightly alkaline soil conditions is assumed to be primarily nitrification.

Emissions of N_2_O have been consistently impacted by source of fertilizer in turfgrass, but these studies did not include PCU [39], [44], [45], [46]. Decreased overall emissions of N_2_O observed in our study with PCU compared to urea have been reported in other crops fertilized with PCU as an alternative N source—finding lower N_2_O emissions with PCU compared to uncoated urea [36],[47]. Decreased emissions of N_2_O from fertilization with PCU compared to urea over the course of a growing season also have been documented in barley (*Hordeum vulgare* L.), cabbage (*Brassica oleracea* L.), maize (*Zea mays* L.), and potato [27], [38], [48], [49]. Hyatt et al. (2010) reported overall reductions of N_2_O emissions from PCU of 39% compared to urea in potato on coarse-textured soils-values that are within the range of reduction observed in the present study [27].

This study was conducted during the fall season to an established sod at a time of relatively low temperatures that steadily declined with time. Such late season fertilization of N is common and applying a majority of the N at this time is physiologically beneficial to cool season turfgrass species [50], which are the reasons for implementing the timing schedule in this study. Additional work is also needed to evaluate N_2_O evolution following PCU application to cool season turfgrass earlier in the growing season.

### Nitrate

Although NO_3_^-^ leaching data trended towards less concentration for PCU compared to urea, these differences were not significant from either Site 1 or 2 (*P* = 0.66 and 0.52, respectively). Although timing of irrigation was different between the sites, both were irrigated to mostly match evapotranspiration losses and, therefore, leaching losses would be expected to be minimal and in agreement with our data. If the sites had been over-irrigated, which is common in turfgrass ecosystems, we would expect nitrate leaching differences to have occurred as has been observed in previous studies using PCU in turf and other cropping systems [30], [31], [32].

### Nitrogen Uptake and Verdure

Growth and verdure improvements were observed in turfgrass grown with either urea or PCU compared to the turfgrass grown without N application (control). At both sites there were no observable differences in verdure or growth between PCU and urea. The average visual rating of turfgrass grown with application of PCU and urea were statistically similar and averaged 4.5 and 3.8 at Site 1 and 2, respectively. Both PCU and urea visual ratings were significantly better than the control, which averaged 2.5 at both locations. This response is typical for turfgrass.

Shoot total N concentrations from samples removed on DOY 292 at Site 1 varied significantly among treatments (Table 2). Specifically, N concentration in turfgrass was maintained significantly higher in the order of urea > PCU > control. However, no other root and shoot samples taken at other times or sites were significantly different in N concentration (Table 2). The lack of treatment effects at Site 2 may have been related to its history as an established area of turfgrass with regular N application, leading to a large organic N pool in constant cycling with consummate higher background losses of N (Table 1). A more rapid release of N to turf from urea compared to PCU explains higher N content from urea application at Site 1. In general, even no application of N maintained N content in or slightly below the low range of N considered adequate for shoot N levels, i.e. 2.51–5.10% in Kentucky bluegrass and 3.34–5.10% in perennial ryegrass [51]. As has been found with other crops [18], [21], [28], PCU provides ample N to the plant while mitigating environmental losses of NH_3_ and N_2_O.

**Table 2:** Root and shoot N concentrations of a mixed stand of Kentucky blue grass and perennial rye grass sampled at one in-season and one end-of-season date for each of two sites.

Location	Sampling date, Julian day	N source	- - - - - - - - - - - N^†^, % - - - - - - - - - - - -	
			Roots	Shoots
Site 1	292	Control	-	2.7 C
		PCU	-	4.2 B
		Urea	-	5.1 A
	340	Control	0.4 A	3.9 A
		PCU	0.8 A	3.2 A
		Urea	0.7 A	3.1 A
Site 2	316	Control	-	3.7 A
		PCU	-	3.1 A
		Urea	-	4.0 A
	340	Control	0.6 A	3.3 A
		PCU	0.6 A	2.7 A
		Urea	0.6 A	2.5 A

^†^ For comparing N sources for a given site and date of sampling, values with the same letter are not significantly different at *P* = 0.05.

### Conclusions

Our findings indicate the health and appearance of the cool season turfgrass mixture of Kentucky bluegrass/perennial ryegrass can be maintained by utilizing PCU and at the same time mitigate environmental losses as N_2_O and NH_3_. For example, urea resulted in 127–476% more measured N_2_O and 121–368% more measured NH_3_ impact on the environment than PCU or the control, respectively. Our research identifies no downside to PCU use under these conditions. Reductions of the magnitude reported herein of N_2_O [this long-lived (150 year stratospheric life), potent (310 times the GWP of CO_2_) greenhouse gas] deserve further investigation by longer term studies under various environmental conditions in all fertilized landscapes.

## Supporting Information

S1 Dataset Raw data collected at both sites for NH4^+^, N_2_O, NO3^-^, tissue and weather presented in spreadsheet form. (XLS)
